# Impact of Botulinum Toxin Injections on Quality of Life of Patients with Long-Standing Peripheral Facial Palsy

**DOI:** 10.3390/toxins16030140

**Published:** 2024-03-08

**Authors:** Jérémy Amar, Frédéric Tankere, Diane Picard, Lauranne Alciato, Fabienne Carré, Claire Foirest

**Affiliations:** ENT Department, Pitié Salpêtrière Hospital, Sorbonne Université, 75013 Paris, France; frederic.tankere@aphp.fr (F.T.); diane.picard@aphp.fr (D.P.); lauranne.alciato@aphp.fr (L.A.); fabienne.carre@aphp.fr (F.C.); claire.foirest@aphp.fr (C.F.)

**Keywords:** long-standing facial palsy, botulinum toxin, quality of life, hemi-facial spasm 30, HFS 30, facial clinimetric evaluation (FaCE)

## Abstract

(1) Background: Sequels of facial palsy lead to major psychosocial repercussions, disrupting patients’ quality of life (QoL). Botulinum toxin (BoNT) injections can permit us to treat long-standing facial palsy, improving facial symmetry and functional signs including synkinesis and contractures. (2) Methods: The main aim of this study was to assess the evolution of the QoL for patients with long-standing facial palsy before, at 1 month, and at 4 months after BoNT injections by using three questionnaires (HFS-30, FaCE, and HAD). The other goals were to find clinical factors associated with the improvement in the QoL and to assess the HFS-30 questionnaire for patients with unilateral facial palsy (3) Results: Eighty-eight patients were included in this study. There was a statistically significant improvement in QoL at 1 month after injections, assessed using the three questionnaires. This improvement was sustained at 4 months after the injections, with a statistically significant difference for the HFS-30 and FaCE questionnaires. (4) Conclusions: This study showed that the BoNT injections lead to a significant increase in the QoL of patients with unilateral facial palsy. This improvement is sustained 4 months after the injections.

## 1. Introduction

Peripheral facial palsy (PFP) is a relatively frequent condition that is caused by various etiologies [1]. The main causes are Bell’s palsy (60–80% of cases), post-traumatic facial palsy, neoplasms, or Zoster infections [2,3].

Although facial palsy is recovered from completely in most cases, sequelae can persist in 20 to 30% of cases [1]. PFP often leads to residual facial hypotonia and denervation phenomena, persisting even beyond the acute stage.

In severe cases (House and Brackmann grade V or VI [3]), affected muscles are less tense, causing collapsing in the affected regions and asymmetry during both rest and movement. Clinical signs include brow ptosis, exacerbating pre-existing dermatochalasis and potentially affecting the visual field during lateral looking. Weakness in the orbicularis oris muscle results in asymmetrical lip movements and a drooping labial commissure, leading to issues like salivary incontinence and speech disturbances [4,5]. 

Post-paralytic syndrome, which occurs between the 5th and 10th months, is due to aberrant axonal reinnervation. Tonic complications manifest as spastic sequelae, often observed after acute episodes of PFP. Synkinesias, involuntary movements during voluntary muscle action, are common (40–70% of sequelae) and affect various facial muscle groups [6,7,8]. Spasms, involving involuntary contractions, and hyperactivity on the contralateral side can also emerge, causing functional and aesthetic challenges. For example, frontalis hyperactivity may accentuate forehead wrinkles, impacting the visual field. Lagophthalmos, ectropion, and complications like chronic irritation and keratitis further contribute to the complexity of PFP sequelae. Secretory complications result from aberrant reinnervation of parasympathetic fibers, leading to conditions like “crocodile tears” and Frey syndrome [6,7,9]. Understanding the diverse complications associated with PFP sequelae is crucial for comprehensive patient care and underscores the need for tailored interventions to treat both functional and aesthetic aspects [4,5,6].

Treatment of this condition is based on specific facial reeducation, botulinum toxin (BoNT) injections, and, in some cases, surgical procedures. 

Because of its dose-dependent and reversible action in blocking neuromuscular transmission, BoNT is an integral part of the therapeutic tools that are used to treat the sequelae of PFP. BoNT injections have revolutionized treatment outcomes, focusing on improving facial symmetry by treating contractures, synkinesias, and contralateral hypertonia [10,11]. Anatomical and functional knowledge of the muscles of the face and neck is therefore essential for any physician wishing to perform this type of injection. 

However, injections are generally delayed until the sixth month to minimize the risk of exacerbating sequelae, particularly synkinesias [12]. Peripheral facial muscle contractions induce physiological tissue movements, contributing to wrinkles and furrows, which is crucial to understanding and treating the sequelae of paralysis [5,12,13]. 

It is important to know the agonist/antagonist functions of the facial muscles, in order to best treat the sequelae. Indeed, a palpebral occlusion may be weakened by aberrant contracture of the homolateral frontal muscle during the voluntary eye-closure movement. As the frontalis muscle is antagonistic to the orbicularis muscle, it should relax to give the orbicularis muscle all the strength that it needs for effective palpebral occlusion. Some patients with aberrant reinnervation sequelae may therefore have weakened palpebral occlusion. Similarly, on the lower level of the face, when smiling, the labial commissure depressor muscle must relax to allow the elevator muscles to come into action (notably, the zygomaticus major and minor). Paradoxical contractures may occur in the event of sequelae, with downward traction of the labial commissure, even though the elevator muscles may have retained hypotonic sequelae. The resulting smile then takes on the appearance of a sigmoid smile, which can be treated, at least partially, by fine, targeted injections of botulinum toxin [4]. 

A personalized injection plan is crucial, taking into account the evaluation of each muscle at rest, during facial expressions, and at maximum contraction [12]. Individual variations in injection points and doses depend on the severity of sequelae, patient expectations, and levels of discomfort. It is recommended to gradually increase the dose over the course of sessions in order to assess the patient’s sensitivity and minimize the risk of discomfort or perceived regression [13]. Close monitoring after injection, particularly between the second and fourth week, enables adjustments to be made and ensures the patient’s comfort and the treatment efficacy [12,13]. This comprehensive approach ensures the optimal use of BoNT injections in the management of PFP sequelae.

These esthetic and functional symptoms are responsible for major social and psycho-affective repercussions, with significant repercussions in body image and socio-professional interactions [14]. The psychosocial and aesthetic consequences of PFP are substantial and often underestimated. Individuals with facial paralysis may experience a decline in self-esteem, social withdrawal, and increased levels of anxiety and depression [15]. The altered facial appearance can result in a negative perception by others, leading to stigmatization and a sense of isolation. Facial asymmetry, drooping mouth, and an inability to convey emotions through facial expressions contribute to a diminished quality of life for these patients [8,9,10]. 

PFP has a profound impact on patients, leading to psychological distress [16] and social impairment [17]. Anxiety and depressive symptoms may affect up to 50% of patients with PFP [18], but the psychosocial impact has not been sufficiently studied. 

Epidemiological studies [10], including data from 57,941 patients, have revealed that up to 9.7% of adults and 6.4% of children with PFP will develop a depressive state within two years of diagnosis. Women over 40 appear to be particularly vulnerable to this risk [11]. Higher rates of anxiety and depressive symptoms have also been reported in smaller studies [11,18].

Despite wide recognition of the psychosocial impact of PFP, the existing literature often presents methodological limitations, highlighting the need for further exploration [15]. Psychological distress should be considered a crucial element of integral patient management, given that rehabilitation strategies aim not only to restore function, but ultimately to improve a patient’s QoL.

Several validated scales [19], including the Clinical Facial Evaluation (FaCE) [20] and the Facial Disability Index (FDI) [21], assess the quality of life of patients with PFP [17,22,23,24,25,26,27]. However, results concerning the correlation between psychological distress and clinical severity are discordant, and some report no systematic relationship between psychological suffering and the clinical grade of PFP [18,22], while others establish significant associations between QoL scores and the severity of facial palsy [24,28].

Tavares-Brito et al. have identified factors such as overweight, anxiety, chronic pain, prior treatment, radiotherapy, and the duration of PFP progression as contributing to a poorer QoL. In addition, female gender [26,28,29,30], age [26], and the presence of perioral sequelae [31] were associated with a significative negative impact on QoL.

Few studies explore the impact of BoNT on the quality of life of PFP patients, with varying results. A systematic review was conducted by Luijmes et al. [32] to investigate the effect of PFP on the QoL before and after different treatment modalities and reported two studies assessing the QoL after BoNT injections [33,34]. Another literature review conducted by Fuzi et al. [35] found six articles assessing the quality of life after BoNT injection [14,26,33,34,36,37]. These studies highlighted an improvement in the QoL after BoNT injection. However, most of these studies were carried out in a limited cohort and/or with a short follow-up period. The FaCE and FDI scales, recently translated to and validated in French [38], are widely used to assess quality of life. The Hemifacial Spasm 30 (HFS-30) questionnaire, validated for essential hemifacial spasm [39], and also recently validated in French [13], shows potential applicability to PFP due to shared functional impairment and socio-professional challenges.

The aim of this study was to assess the impact of BT injections on the QoL of patients presenting with sequelae of facial palsy. 

The first aim of this study was to assess the quality of life of patients (QoL) before and at 1 and 4 months after the injection. The subsequent aims were to identify the potential clinical outcomes associated to the improvement in the QoL and to assess the applicability of the Hemi-Facial Spasm 30 questionnaire in patients with peripheral facial palsy PFP.

## 2. Results

### 2.1. Demographic and Clinical Data

Sixty-three women (72%) and twenty-five men (28%) were included. The mean (SD) age was 53 (15.8) years. In most cases, the facial palsy was idiopathic (55%). Among the post-traumatic facial palsy cases, we found 12 cases post acoustic neurinoma surgery and 8 cases resulting from different causes (ponto-cerebellous meningioma resection, petrous apex paraganglioma, cochlear implant, post-cervical wound, and after cholesteatoma surgery). In six cases, the etiology was mixed, tumoral, and traumatic, with tumoral facial palsy that was worsened by surgery (three cases of facial nerve schwannoma, two cases of facial hemangioma, and one case of parotid tumor). Four cases of facial palsy included a general disease (Gougerot–Sjoren, Waldenström, granulomatosis with polyangeiitis, and cochleo-vestibular syndrome), and one case was secondary to meningitis.

The initial House and Brackman score was V and VI in, respectively, 48% and 34% of cases. The delay since the last injection at the inclusion was 7.2 ± 3.39 months. The PFP occurred 5.7 ± 7 years ago at the point of inclusion in the study. Latest Sunnybrook score before injection was 62.9 ± 33. All the demographic data are summarized in Table 1.

### 2.2. Longitudinal Evolution of QoL Questionnaires 

#### 2.2.1. Total Scores 

**Figure 1:**
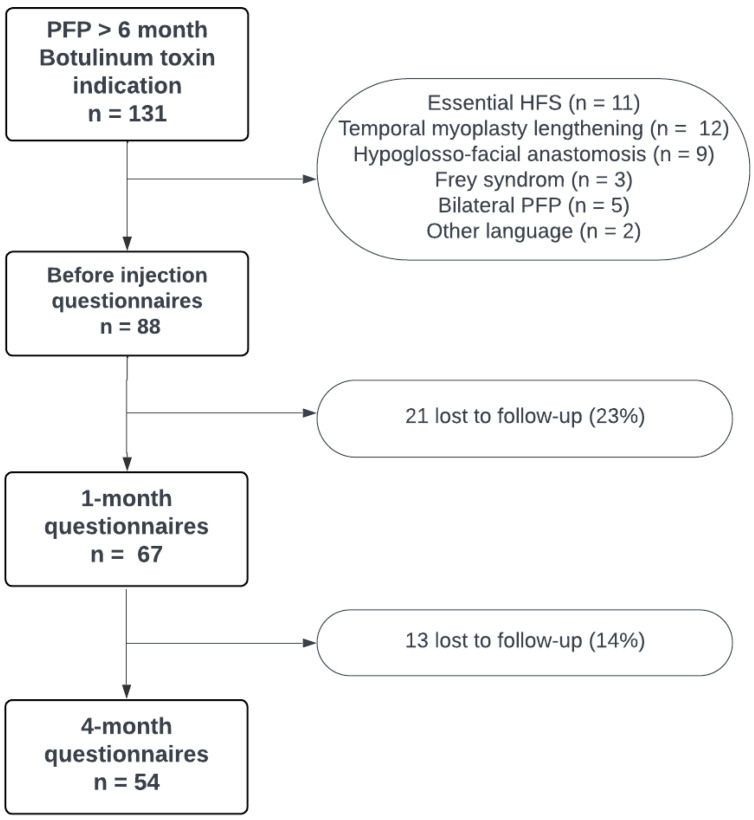
Flowchart of included, excluded, and lost to follow-up patients.

Regarding the questionnaires, 88 patients answered the three questionnaires at the time of the inclusion. Sixty-seven patients answered the 1-month questionnaires (76%), and fifty-four answered the 4-month questionnaires (61%). The number of participants at each step is summarized in Figure 1.

The results of the total scores before and at 1 month and 4 months after BoNT injections were statistically significant for the three questionnaires at 1 month and for the HFS-30 and FaCE at 4 months. The results are summarized in Table 2.

For the HFS-30 and the FaCE questionnaires, we found a statistically significant difference between the score before and at 1 month after injection with *p* < 0.0001 (respectively, for HFS-30 and FaCE, t = 4.7; df = 66 and t = 4.4 df = 66). The difference between the score before and at 4 months after injection was also statistically significant for the two questionnaires (respectively, for HFS-30 and FaCE *p* = 0.94; t = 0.08; df = 49 and *p* = 0.0003; t = 3.9; df = 53). 

For the HAD scale, there was also a statistically significant difference between the score before and at 1 month after injection (*p* = 0.0032; t = 4.7; df = 66), but no difference was found between the mean total scores, between the questionnaire before injection, and at 4 months (*p* = 0.38; t = 0.87; df = 53).

For the three questionnaires, no statistically significant differences were found between the score at 1 month and at 4 months.

**Figure 2:**
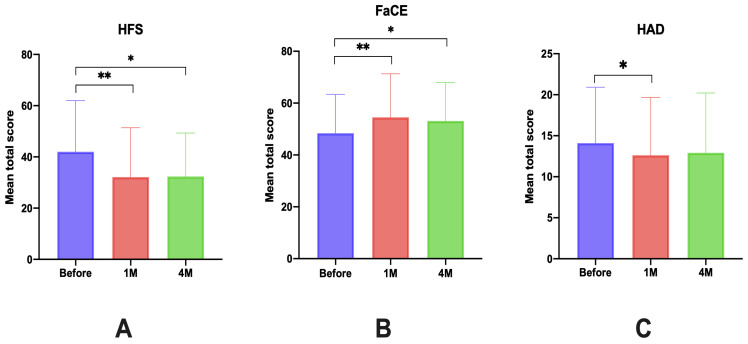
Mean total score before and at 1 month and 4 months after injections for the three questionnaires. (**A**): HFS-30; (**B**): FaCE; (**C**): HAD; * *p* < 0.05; ** *p* < 0.001.

The global results of the obtained scores before and at 1 month and 4 months after injections are summarized in Figure 2.

#### 2.2.2. Total Score by Domains

The domains of the HFS-30 and FaCE scores are summarized in Table 3 and Table 4.

For the HFS-30 questionnaire, there was a statistically significant improvement at 1 month post-injection for almost all questionnaire domains. The improvement was highly significant (*p* ≤ 0.001) for the “well-being” (*p* = 0.0001; t = 4.0; df = 66) and “stigma” (*p* < 0.0001; t = 5.5; df = 66) domains. The improvement was significant (*p* < 0.05) for “mobility” (*p* = 0.0021; t = 3.2; df = 66), “daily living” (*p* = 0.040; t = 2.0; df = 66), “cognition” (*p* = 0.0049; t = 2.9; df = 66), and “communication” (*p* = 0.0014; t = 3.3; df = 66). There was no significant difference in the “social support” domain.

Regarding the FaCE questionnaire, the domains where the difference was highly significant (*p* ≤ 0.001) were “facial comfort” (*p* < 0.0001; t = 4.2; df = 66), “social function” (*p* = 0.0002; t = 3.9; df = 66), and “oral function” (*p* = 0.0010; t = 3.4; df = 66). There was also a statistically significant difference for the “eye comfort” domain (*p* = 0.0128; t = 3.4; df = 66). There was no significant difference in the “tear control” and “facial movement” domains.

#### 2.2.3. Subgroup Analyses

##### Qualitative Data

We compared the difference between responses to the FaCE and HFS-30 questionnaires before and 1 month after injection in different subgroups. The results are summarized in Table 5.

We found a statistically significant difference between men and women, with an average differential score of 1.19 for men and 12.54 for women for the HFS-30 questionnaire (*p* = 0.0164; t = 2.55; df = 66).

The same trend was found for the FaCE questionnaire, with a differential score of 1.35 for men and 8.16 for women, but this difference was not statistically significant (*p* = 0.08; t = 1.82; df = 66).

There was also a statistically significant difference according to the number of previous injections for the HFS-30 questionnaire when comparing patients who had never had an injection (differential score at 17.5) with patients who had already had three or more injections for the HFS-30 questionnaire (mean ∆ differential score of 17.5 for 0 injections and 6.83 for ≥3 injections; *p* = 0.0458; t = 2.03; df = 66).

No significant differences were found when comparing the different subgroups within the FaCE questionnaire.

##### Quantitative Data

We analyzed the correlations in the quantitative data by comparing them individually against the differential ∆ score “pre-injection–1 month post-injection”.

For the analysis, we selected the data that seemed to be relevant. Correlation was analyzed for age, BMI, Sunnybrook, sum of reported sequelae (if a sequela was present, it counted as 1 point out of a total of 9 sequelae listed), and duration of PFP evolution. The results are summarized in Table 6.

We found a statistically significant correlation between patient age and the difference between pre-injection and 1 month post-injection (∆ score), with a negative correlation for the HFS-30 questionnaire (r = −0.46; *p* < 0.0001) and a positive one for the FaCE (r = 0.27; *p* = 0.03). The younger the patients were, the more improved they were at 1 month post-injection according to the HFS-30 and FaCE questionnaires.

A negative correlation was found for the HFS-30 between the duration of PF evolution and the ∆ score, meaning that a longer evolution of PF is associated with a poorer improvement in QoL.

## 3. Discussion

Long-standing PFP significantly impacts patients’ QoL [13,14,15,16], emphasizing the critical need for understanding and managing these sequelae through surgical and non-surgical treatments. BoNT plays a crucial role in managing these long-term sequelae [11,12,17].

The study’s notable strength lies in its substantial patient cohort, a rarity in prospective studies specifically assessing QoL post-BoNT injection (only seven articles were found [14,26,27,33,34,36,37], with the largest cohort being 66 patients in the Mehta and Hadlock study [33]). The rate of questionnaire non-participation at 1 (24%) and 4 months (39%) can induce a potential selection bias. While no significant differences were found in the pre-injection questionnaire scores between participants and those who were lost to follow-up, potential dissatisfaction among the latter group cannot be ruled out.

The study chose a limited questionnaire approach for better participation rates, comparing FaCE to HFS-30 due to its prevalence in the literature [11,12,13,14,40]. Additionally, the HAD scale was selected for its ease of use and wide representation in facial paralysis studies to assess the psychosocial impact [19,20,21].

Despite PFP’s primary impact on a patient’s QoL, research examining therapeutic impacts on QoL remains rare [7,22,23]. This study is to the best of our knowledge the first French assessment of QoL post-injection in PFP patients since the FaCE questionnaire’s translation by Barry et al. [38]. Demographic similarities with Tavares-Brito et al.’s [24] extensive cohort validate the study’s representation of PFP patients (an average age of around 50, with a majority of women included in the study and a similar etiological distribution). The initial score on the FaCE questionnaire was also similar to that found in our study (47.6 vs. 48.4 in our study).

The analysis in our study reveals no significant correlation between injection parameters and QoL improvement, emphasizing individualized treatment planning. There was therefore in our study no relationship between the quantity of toxin that was injected and the improvement in quality of life [24].

Mehta and Hadlock’s [33] study evaluated the QoL of 66 patients, using the FaCE questionnaire before and 10 days after injections. We reported a similar pre-injection FaCE score (51.7 versus 48.4 in our study), but a substantial FaCE score increase at 10 days post-injection when compared to the 1-month score in our study, which seemed to us to be the optimum time for obtaining the best results after injection (63.7 at 10 days versus 54.4 at 1 month in our study). However, missing data and a lack of injection history details in that study should raise suspicions about potential bias.

The study observed a significant QoL improvement across various questionnaire domains, except in “social environments” in the HFS-30. This is probably due to the fact that there is a double-negative formulation in questions 19 and 20 of the French questionnaire which is probably misunderstood by patients, which shows that the French translation of this questionnaire may still need to be improved.

In the FaCE questionnaire, the improvement in “lacrymal control” was not significant, probably due to a lack of patients with a lacrymal complaint in the cohort. The improvement in “facial movement” was non-significant too, probably because the improvement is not so much in movement recovery but in facial comfort through the treatment of contractures and synkinesis.

The HAD scale analysis highlighted significant anxiety and depressive symptoms among PFP patients, which is consistent with the existing literature [19,20,21].

Despite its non-specific nature, the HAD scale demonstrated notable improvement post-injection, although it was less pronounced compared to other questionnaires (HFS-30 and FaCE).

At 4 months, this improvement was no longer significant. This persistence of QoL enhancement beyond the expected duration of BoNT effects suggests a potential remnant effect, warranting reassessment at later intervals.

Individual variability in response to BoNT implies a minimum four-month interval between injections, but personalized adjustments may be necessary based on each patient’s response. A routine inquiry about patients’ preference for earlier injection sessions could help in individualized treatment scheduling. These findings underline the need for tailored follow-up intervals, considering both the persistent effects of the toxin and individual patient responses, thus optimizing PFP management strategies.

Younger age and female gender correlated with greater QoL improvement post-injection, which is in line with the existing literature [16,30]. It would therefore seem that women are more affected by PFP than men, and therefore more in need of treatment for the sequelae. This would also explain why a majority of women were included in the study (72%).

Concerning the study of subgroups after injections, Shinn et al. [30] found similar data to our study. In a cohort of 99 patients injected and re-evaluated using the Synkinesia Score (SAQ), they found, as in our study, an association between the degree of improvement, young age, and female gender.

Younger women would therefore be more affected by the sequelae of PFP, but also more sensitive to the effects of botulinum toxin injections.

Furthermore, only the HFS-30 questionnaire showed a significant difference between men and women. This suggests that this questionnaire is more sensitive than the FaCE is for assessing inter-group differences. These results could be explained by the fact that women, particularly younger women, are more affected by facial asymmetry in their social relationships than their older male counterparts are. The latter would have learned to live with this paralysis and would therefore have lower demands with regard to injections.

Synkinesis sequelae seemed to significantly impact psychosocial aspects, warranting further investigation involving the correlation between the QoL and SAQ questionnaires.

Surprisingly, patients receiving fewer injections showed more significant QoL improvement initially, possibly due to the novelty of treatment effects. However, a prolonged PFP duration correlated with reduced post-injection improvement, which is consistent with other studies [16,24]. This result might suggest that sequelae that have settled in after a long duration of PFP evolution would be more difficult to treat with toxin in these patients.

No correlation was found between the QoL scores and the House and Brackmann and Sunnybrook scales.

Bylund et al. [28] evaluated the evolution of the QoL in patients with PFP during follow-up and its correlation with the House and Brackmann and Sunnybrook classifications. In this study, the total FaCE score that was found at more than 6 months after the start of PFP was higher than in our study (66 on average versus 48.4 in our study) for a similar Sunnybrook score (64 versus 63 in our study). Data concerning the domains of the FaCe score were also similar to our study. In the Bylund study, there was a strong correlation between the FaCE questionnaire and the Sunnybrook classification. This correlation was not found in our study. This may be due to the large number of missing data concerning Sunnybrooks in our study, as well as the fact that their evaluation sometimes predated the day on which the questionnaire was filled (the last available Sunnybrook score was recorded in our study when it was notified in the medical record).

Concerning the results of the post-injection questionnaires, few similar studies were found in the literature [14,30,32,33,34,35,37], and these always concerned small cohorts of patients. As for the House and Brackmann score, its simplicity and the large number of patients who were classified as grade III made it difficult to assess the efficacity of BoNT injections.

We found no correlation between the initial severity and quality of life. This is probably due to the fact that the majority of patients initially had grade V or VI facial paralysis. This is a poor prognostic factor for recovery and the occurrence of sequelae, but at the sequelae stage, all these patients were equivalent.

The HFS-30 questionnaire differs from the FaCE in that the questions are mainly based on daily life and social impact, whereas the FaCE is more focused on the impact of functional signs specifically. This questionnaire is well validated in the study of the QoL in patients with essential hemifacial spasm [39,40,41] but has never been used for PFP.

The data from our study seem to show that this test is suitable for assessing the QoL of patients with PFP, and it even seems to be more sensitive for discriminating certain subpopulations than the FaCE score.

## 4. Conclusions

This study is the first French study to evaluate the quality of life (QoL) in patients with long-standing PFP after BoNT over a substantial four-month follow-up period. A significant enhancement in QoL was observed within one month across the three tested questionnaires in nearly all domains. Female gender and younger age were associated with more pronounced QoL improvement, while prolonged PFP duration correlated with less enhancement. Notably, the enduring impact of injections on QoL persisted even at the four-month post-injection evaluation.

Moreover, the HFS-30 questionnaire, previously validated for essential hemifacial spasm, seems suitable for assessing the QoL among PFP patients. These findings underscore the beneficial effect of botulinum toxin injections on individuals with sequelae of facial palsy. Given the substantial psychosocial impact of facial paralysis, a QoL evaluation should routinely accompany functional assessments during patient evaluations. Regular QoL assessments using diverse questionnaires could aid in individualized determination of optimal intervals between botulinum toxin injections.

While these encouraging findings indicate the need for further exploration in larger cohorts, cross-referencing with severity scores (House–Brackmann or Sunnybrook) or functional scores (Synkinesis Assessment Questionnaire) in future studies would validate these outcomes.

## 5. Materials and Methods

We led an observational, prospective, monocentric study based on 88 patients presenting with long-standing unilateral facial palsy with a planned treatment of botulinum toxin injection. Three questionnaires were submitted to patients: the Facial Clinimetric Index (FaCE) [20], the Hemi-Facial Spasm 30 (HFS 30) [21], and Hospital Anxiety and Depression (HAD) [42].

### 5.1. Population

We included all patients with unilateral long-standing (>6 months) PFP with synkinesis or post-paralytic hemi-facial spasm with botulinum toxin injection planned (first injection or not). The indication of the injection was previously decided, independently of the study, and all the injections were performed by the same operator in our university ENT department, who was blinded to the included patients. We excluded patients with essential hemi-facial spasm, bilateral facial palsy, or associated Frey’s syndrome. Patients with a medical history of lengthening temporal myoplasty of hypoglosso-facial anastomosis and patients younger than 18 years old or unable to understand the questionnaires were also excluded. Eighty-eight patients were included in this study between November 2021 and August 2022.

All patients gave their informed and written consent according to the IRB approval number 20211130113517 (APHP registery).

### 5.2. Data Collection

The following data were collected on the medical records: demographics (gender, age, body mass index (BMI)), previous medical history, surgical history (otologic surgery, vestibular schwannoma, facial schwanoma, orbito-palpebral surge), data about the facial palsy medical history (date of onset, side, etiology, initial House and Brackman grade, initial treatment by corticoids or antiviral drugs), data about the facial palsy actual sequelae (synkinesis, hemi-facial spasm, myokimia, residual hypotonia, gusto-lacrymal syndrom), last reported Sunnybrook score [43] in medical records, and data about the injections (number of anterior injections), the type of botulinum toxin used (Botox^®^ or Xeomin^®^), the total number of injected unit in each side (paralyzed and healthy side).

The questionnaires were submitted after providing information to and obtaining consent from patients, before the injection. The 1- and 4-month questionnaires were collected by mail.

### 5.3. Statistics

All the statistical analyses were performed using JMP^®^ software (Version 16.2 SAS Institute Inc., Cary, NC, USA, 1989–2022). To assess the evolution of QoL before and at 1 and 4 months after the injections, we compared the total score of the three questionnaires using a paired Student’s test after checking the normality by using a Kolmogorov–Smirnov test. For the FaCE questionnaire, the total score was normalized between 0 and 100.

The domains of the FaCE and HFS 30 questionnaires were compared by using a paired Student’s test.

The different clinical outcomes which seemed to be relevant were assessed using Student’s tests for qualitative data by comparing the ∆ score (difference between the score before and after injection). Pearson’s correlation coefficient was used to compare the quantitative data. A two-by-two correlation analysis using a linear regression model was carried out for the continuous quantitative data, comparing them individually against the differential ∆ score “pre-injection–1 month post-injection”.

## Figures and Tables

**Table 1:** Demographic and clinical data.

Clinical Data	Value
Men (n, %)/Women (n, %)	25 (28%)/63 (72%)
Age (mean ± DS [rank])	53 ± 15.8 [26–90]
PFP etiology (n, %)	
- Idiopathic	49 (55%)
- Zoster	8 (9%)
- Traumatic	20 (23%)
- Tumoral and traumatic	6 (7%)
- General disease	4 (5%)
- Other	1 (1%)
Initial HBS	
- III (n, %)	2 (2.2%)
- IV (n, %)	4 (4.5%)
- V (n, %)	43 (48%)
- VI (n, %)	30 (34%)
Actual Sunnybrook score (mean ± DS)	62.9 ± 33 [17–91] (*n* = 40)
Nb of months since last injection	7.2 ± 3.39 [2.8–22.3]
Mean ± SD [range]	
Nb of years since the beginning of PFP	5.7 ± 7 [0.45–45]
Mean ± SD [range]	

Nb = number; HBS = House and Brackman Score; SD = standard deviation.

**Table 2:** Total scores of the three questionnaires before and at 1 month and 4 months after injection.

Questionnaire (Mean ± SD)	HFS-30			FaCE			HAD		
Delay after Injection	Before	1 Month	4 Months	Before	1 Month	4 Months	Before	1 Month	4 Months
Mean value (±SD)	42.0 ± 20.0	32.1 ± 19.2	32.4 ± 17.0	48.4 ± 15.0	54.4 ± 16.8	53.1 ± 14.5	14.1 ± 6.8	12.7 ± 7.0	12.9 ± 7.3
∆ Score ^1^		9.9	9.6		−6.1	−4.7		1.4	0.78
*p*		<0.0001 **	<0.0001 **		<0.0001 **	<0.003 **		0.0032 *	0.38

SD = standard deviation; * *p* < 0.05; ** *p* < 0.001; ^1^ difference between the score before–1/4 months after injection.

**Table 3:** Total scores for each domain of the HFS-30 questionnaire before and at 1 month after injection.

Domains (Mean ± SD)	Before Injection	1 Month	∆ Score ^1^	*p*
Mobility	5.1 ± 4.0	3.9 ± 3.6	1.2	0.0021 *
Activities of daily living	5.2 ± 4.6	4.1 ± 3.8	1.1	0.040 *
Emotional well-being	9.6 ± 5.7	7.0 ± 5.6	2.5	0.0001 **
Stigma	9.5 ± 4.0	6.9 ± 4.0	2.5	<0.0001 **
Social support	3.8 ± 3.3	3.8 ± 3.1	0.05	0.97
Cognition	4.2 ± 2.8	3.3 ± 2.6	0.9	0.0049 *
Communication	4.7 ± 2.8	3.4 ± 2.6	1.3	0.0014 *

SD = standard deviation; * *p* < 0.05; ** *p* < 0.001; ^1^ difference between the score before–1 month after injection. Scores were compared by paired *t*-test.

**Table 4:** Total scores for each domain of the FaCE questionnaire before and at 1 month after injection.

Domains (Mean ± SD)	Before Injection	1 Month	∆ Score ^1^	*p*
Facial movement	40.8 ± 22.2	41.5 ± 21.44	−0.7	0.85
Facial comfort	31.4 ± 22.1	44.0 ± 26.2	−12.6	<0.0001 **
Oral function	60.1 ± 28.7	67.2 ± 25.7	−7.1	0.0010 **
Eye comfort	45.9 ± 28.4	49.4 ± 29.8	−3.6	0.0128 *
Lacrimal control	52.8 ± 31.3	59.3 ± 31.9	−6.5	0.1
Social function	61.3 ± 24.0	68.6 ± 23.5	−7.3	0.0002 **

SD = standard deviation; * *p* < 0.05; ** *p* < 0.001; ^1^ difference between the score before–1 month after injection. Scores were compared by paired *t*-test.

**Table 5:** Comparison of differential score before injection–score at 1 month post-injection according to different subgroups for the HFS-30 and FaCE questionnaire.

	HFS30		FaCE	
	∆ Score ^1^	*p*	∆ Score ^1^	*p*
Male (n = 17)	1.19		−1.35	
Female (n = 50)	12.54	0.0164 *	−8.16	0.08
Orbito-palpebral surgery				
Yes (n = 54)	5.15		−4.10	
No (n = 13)	11.43	0.18	−7.24	0.39
Nb of anterior injections				
0 (n = 16)	17.5		−9.44	
1 (n = 8)	4.25	0.0736 ^2^	−2.50	0.21 ^2^
2 (n = 14)	10.86	0.28 ^2^	−3.80	0.23 ^2^
≥3 (n = 29)	6.83	0.0458 *^2^	−7.13	0.56 ^2^

^1^ Difference between the score before–1 month after injection; ^2^ comparison with the 0-injections group; * *p* < 0.05; scores were compared by *t*-test.

**Table 6:** Correlation between quantitative data and differential score before injection–1 month after injection.

	HFS-30		FaCE	
Clinical Data	r	*p*	r	*p*
Age	−0.46	<0.0001 **	0.27	0.03 *
BMI	−0.028	0.82	0.0038	0.98
Sunnybrook	0.19	0.23	0.13	0.41
Sum of sequelae	0.011	0.93	−0.07	0.58
Duration of PFP	−0.34	0.008 *	−0.21	0.10

Scores were compared by Pearson’s coefficient of correlation (r). * *p* < 0.05; ** *p* < 0.001.

## Data Availability

The data presented in this study are available upon request from the corresponding author. The data are not publicly available.
